# Inequalities in utilization of maternal and child health services in Ethiopia: the role of primary health care

**DOI:** 10.1186/s12913-016-1296-7

**Published:** 2016-02-12

**Authors:** Solomon Tessema Memirie, Stéphane Verguet, Ole F. Norheim, Carol Levin, Kjell Arne Johansson

**Affiliations:** Department of Global Public Health and Primary Care, University of Bergen, Bergen, Norway; Department of Global Health and Population, Harvard T.H. Chan School of Public Health, Boston, MA USA; Department of Global Health, University of Washington, Seattle, WA USA

**Keywords:** Inequality, Maternal and child health services, Primary health care, Ethiopia

## Abstract

**Background:**

Health systems aim to narrow inequality in access to health care across socioeconomic groups and area of residency. However, in low-income countries, studies are lacking that systematically monitor and evaluate health programs with regard to their effect on specific inequalities. We aimed to measure changes in inequality in access to maternal and child health (MCH) interventions and the effect of Primary Health Care (PHC) facilities expansion on the inequality in access to care in Ethiopia.

**Methods:**

The Demographic and Health Survey datasets from Ethiopia (2005 and 2011) were used. We calculated changes in utilization of MCH interventions and child morbidity. Concentration and horizontal inequity indices were estimated. Decomposition analysis was used to calculate the contribution of each determinant to the concentration index.

**Results:**

Between 2005 and 2011, improvements in aggregate coverage have been observed for MCH interventions in Ethiopia. Wealth-related inequality has remained persistently high in all surveys. Socioeconomic factors were the main predictors of differences in maternal and child health services utilization and child health outcome. Utilization of primary care facilities for selected maternal and child health interventions have shown marked pro-poor improvement over the period 2005–2011.

**Conclusions:**

Our findings suggest that expansion of PHC facilities in Ethiopia might have an important role in narrowing the urban-rural and rich-poor gaps in health service utilization for selected MCH interventions.

## Background

There have been impressive increases in total coverage of essential child health services and child survival in developing countries over the last decades [1]. Even though equity has been stated as an important goal within health sectors, substantial disparities in coverage of maternal and child health services and in under-five mortality between rich and poor children have persisted in most low- and middle-income countries [2–5]. Inequalities across socioeconomic groups and by area of residence are important determinants of maternal and child health [6, 7].

Ethiopia has had a substantial progress in reducing under-five mortality rate (from 198 deaths per 1,000 live births in 1990 to 88 in 2011) [8, 9]. Despite gradual improvement in coverage of child health care services, inequality in child mortality and access to care between urban and rural dwellers and across wealth quintiles remain large. Under-five mortality is 114 deaths per 1,000 live births in rural areas and 83 deaths per 1,000 live births in urban areas. The poorest and the richest quintiles had an under-five mortality of 137 and 86 deaths per 1,000 live births, respectively. Among households with a child having either symptoms of pneumonia or diarrhea; 16 % and 22 % of households from the poorest quintile and 62 % and 53 % from the richest quintile sought care from a health care provider, respectively. The low service utilization occurred in the face of an increased risk of diarrhea and pneumonia among children from the poorest quintile [9].

The national health policy of Ethiopia gives strong emphasis to fulfilling the needs of the rural residents, which constitute 84 % of the Ethiopian population. Ensuring universal access to health care is one of the main targets of the national Health Sector Development Program (HSDP) IV (2011–2015) in Ethiopia [10]. An accelerated expansion of primary health care (PHC) facilities [composed of health centers (HCs) and health posts (HPs)] has been undertaken since 2003. In nearly a decade, the number of HPs and HCs in Ethiopia grew by almost six fold to reach 3245 HCs and 16,048 HPs in 2012/2013. Each health post has two health extension workers (HEWs) and so far a total of 34,850 HEWs were trained and deployed nationally with a ratio to population of 1:2301 that surpassed HSDP III target of 1:2500 [10, 11]. The expansion is envisaged as the key strategy to deliver maternal, neonatal and child health interventions especially to the rural and impoverished segments of the population [12]. According to the 5^th^ National Health Accounts in Ethiopia, 34 % of the total health expenditure was household out-of-pocket spending [13]. It is imperative that such expansions contribute to health equity primarily by moving towards universal access. The 2010 World Health Report has identified inefficient and inequitable use of resources as one of the factors that impede rapid movement towards universal health coverage (UHC) [14].

Inequalities in child health and child survival across household wealth quintiles were examined in the 2005 and 2011 Ethiopian Demographic Health Surveys (DHS) and by Barros et al. in their survey-based analysis of inequality in maternal and child health (MCH) in 54 countdown countries [9, 15, 16]. Skaftun et al. has also examined inequalities in child health in Ethiopia [17]. However, assessments done so far lack some critical MCH interventions (such as family planning) and morbidity outcomes (e.g. stunting) and are not examined in light of the rapid expansion of PHC facilities in Ethiopia. Additionally they did not take into consideration use relative to need, therefore were unable to assess inequity in MCH service utilization.

The main objectives of this study were: (1) to measure changes in degree of inequality in utilization of selected MCH interventions and child morbidities over time; (2) to determine factors associated with inequality and inequity in access to care; and (3) to assess the role of expansion of PHC facilities in Ethiopia on inequality and inequity in access to care using 2005 and 2011 DHS conducted in Ethiopia.

## Methods

### Data and variables definition

We used data from DHS conducted in Ethiopia in 2005 and 2011 [9, 15]. The 2005 and 2011 DHS were conducted on a nationally representative sample of 9,861 and 11,654 households, respectively. The sampling design for both surveys was a two-staged stratified cluster sampling that was not self-weighted at national level. The survey participants/households were stratified into urban or rural groups according to their area of residence. Household’s socioeconomic status was measured using household asset data via a principal components analysis. We used the wealth quintiles as a living standard measure in the subsequent modeling.

Utilization of MCH services was selected for analysis. These were binary variables, where a value of 1 was assigned if care was accessed or a value of 0 if care was not accessed. Both prevention and treatment services were included, where we looked at: medical treatment for diarrhea, skilled birth attendance (SBA), measles immunizations and modern contraceptive usage. We used prevalence of diarrhea, cough, fever and stunting in children as morbidity variables.

### Analysis

Inequality in outcomes was measured by calculating a concentration index, where this index quantifies the magnitude of wealth-related inequality that can be compared conveniently across time periods, countries, regions, or other comparators [18]. The paper by Wagstaff et al provides detailed description of concentration index [18]. In our analysis concentration index (C) was computed as twice the (weighted) covariance between the health variable (*h*) and the fractional rank of the person in the living standard distribution (*r*), divided by the mean of the health variable (*μ*) [19] as:

1 $$ C=\frac{2}{\mu }Cov\left(h,r\right) $$

Concentration index is restricted to values between −1 and 1 and has a value of zero where there is no income-related inequality in outcomes. If the variable reflects morbidity or mortality, the concentration index will usually be negative, showing that ill health is more prevalent among the poor. For coverage indicators, the concentration index is usually positive, as these tend to be higher among the rich [19].

Even though concentration index is a measure of income-related inequality in health care utilization, it does not measure the degree of inequity in use since it still includes legitimate income-related differences in use due to differences in need. Therefore, in our analysis, standardization for differences in need for health care in relation to wealth was done using the method of indirect standardization. Standardization adjusts for the need expected distribution as opposed to the observed distribution of use [20]. To proxy need in health care, the following demographic and morbidity variables were used: age and sex of children under-five years of age and age of women in the reproductive age group (as demographic variables), recent episode of diarrhea (as a morbidity variable in children), history of birth in the past five years (as a proxy of need for SBA) and unmet need for family planning (as a need variable for modern contraceptive usage). Wealth quintile, educational attainment of household head, educational attainment of partner, and area of residence were used as non-need correlates of health care utilization (control variables). Only 0.5 % of the households had health insurance coverage, therefore we did not use it as one of the control variable in our analysis [9].

After estimating the need-standardized utilization, inequity can be tested by determining whether standardized use is unequally distributed across wealth quintiles. Inequity could be measured by estimating the concentration index of need-standardized health care utilization, which is denoted as the health inequity index. Alternatively, the health inequity index can be calculated as a difference between the concentration index for actual utilization and need-expected utilization of medical care [20]. A positive (negative) value of horizontal inequity index indicates horizontal inequity that is pro-rich (pro-poor), while an index value of zero shows absence of horizontal inequity.

The decomposition of the concentration index allows the measurement and explanation of inequality in utilization of health care services across income groups. Wagstaff et al [21] has demonstrated that for any linear regression model of a variable, such as health care use, it is possible to decompose the measured inequality into the contribution of explanatory factors. With this decomposition approach, standardization for need as well as explanation of inequity can be done in one step. Consider the following model:

2 $$ {y}_i=\alpha +{\displaystyle {\sum}_j{\beta}_j{x}_{ji}+{\displaystyle {\sum}_k{\beta}_k\;{z}_{ki}+{\varepsilon}_i,}} $$

, where *x*_*j*_ denotes the need standardizing variables, that includes demographic and health status/morbidity factors, and *z*_*k*_ denotes the non-need variables including socioeconomic status, education, area of residence (urban vs. rural). *α*, *β* and *ε* are the constant, regression coefficients and the error term respectively. The concentration index (C) for utilization of health care can then be written as:

3 $$ \mathrm{C}={\displaystyle {\sum}_j\left({\beta}_j{\overline{x}}_j/\mu \right)}{C}_j+{\displaystyle {\sum}_k\left({\beta}_k{\overline{z}}_k/\mu \right){C}_k+\raisebox{1ex}{$G{C}_u$}\!\left/ \!\raisebox{-1ex}{$\mu \kern0.5em ,$}\right.} $$

, where *C*_*j*_ and *C*_*k*_ are the concentration indices for the need and non-need variables respectively while *μ* is the mean of our health variable of interest (y), $$ {\overline{x}}_j $$ is the mean of *x*_*j*_ and $$ {\overline{z}}_k $$ is the mean of *z*_*k*_. The components $$ \left({\beta}_j{\overline{x}}_j/\mu \right) $$ and $$ \left({\beta}_k{\overline{z}}_k/\mu \right) $$ are simply the elasticity of y with respect to *x*_*j*_ and *z*_*k*_, respectively, that are evaluated at the sample mean. The last term in the equation $$ \left(\raisebox{1ex}{$G{C}_u$}\!\left/ \!\raisebox{-1ex}{$\mu $}\right.\right) $$ captures the residual component that reflects the inequality in health that is not explained by systematic variation across income groups in the need and non-need variables.

Decomposition for non-linear models can only be applied using linear approximation which can introduce errors and is complex. Therefore, even if our health variable of interest is a binary variable, we used the linear model. It has been found elsewhere that decomposition results differ little between ordinary least squares and non-linear estimators [22].

Time trends for changes in mean levels of MCH service utilization were assessed using logistic regression model. MCH service utilizations were used as dependent variables while time of survey as independent variables. We computed the percentage change in excess risk by subtracting one from rate ratio (rate ratio-1), where rate ratio is the incidence in the poorest quintile divided by incidence in the richest quintile (Q1/Q5) [23].

Data were analyzed using the statistical software package STATA (version 13), taking into account the sampling design characteristics of each survey.

### Ethical considerations

We did the analyses using publicly available data from demographic health surveys. Ethical procedures were the responsibility of the institutions that commissioned, funded, or managed the surveys. The study was approved by Regional committees for medical and health research ethics (REK) in Norway and Ethiopian Health and Nutrition Research Institute (EHNRI) scientific and ethical review committee.

## Results

Utilization of measles immunization and modern contraceptive methods has on average increased between 2005 and 2011 (Table 1). Pro-poor coverage changes with a clear dominance were observed for both interventions, demonstrated by significantly (non-overlapping 95 % CI) lower concentration indices in 2011 as compared to 2005. Use of modern contraceptive methods had the widest coverage gap between the poorest and wealthiest in all surveys. In 2011, modern contraceptive methods use rates were 6 % and 44 % for the poorest and the wealthiest quintiles, respectively.

**Table 1:** Average, first and fifth quintile values and concentration indices of selected maternal and child health indicators in Ethiopia (DHS: 2005 and 2011)

Variables	Average value (%)		Lowest quintile value (%)		Highest quintile value (%)		Concentration index (95 % CI)	
Year of DHS	2005	2011	2005	2011	2005	2011	2005	2011
Health service utilization:								
Measles immunization	34.9	55.7*	24.9	45.3	52.5	79.7	0.113 (0.096–0.130)	0.085 (0.074–0.096)
Use of modern contraceptive method^a^	17.4	18.7*	2.6	6.4	36.0	43.5	0.405 (0.369–0.441)	0.275 (0.247–0.303)
Morbidity:								
ARI prevalence in children <5 years	15.9	19.7*	14.8	20.7	13.5	17.0	−0.004 ((−0.015)–0.023)	−0.010 ((–0.048)-0.049)
Diarrhea prevalence in children <5 years	17.2	15.0*	15.6	16.2	14.1	11.3	−0.037 ((-0.067)–(−0.007))	−0.029 ((−0.117)–0.059)
Fever prevalence in children <5 years	17.6	19.3^**^	16.3	21.5	15.2	16.5	−0.025 ((−0.076)–0.026)	−0.025 ((−0.099)–0.049)
Prevalence of Stunting in children <5 years	47.0	44.0*	49.9	47.2	38.4	27.6	−0.026 ((−0.040)–(−0.012))	−0.048 ((−0.062)–(−0.038))

Note: *Indicate the p-value for trend over the period 2005 and 2011 is <0.001. ^**^Indicate the p-value for trend over the period 2005 and 2011 is <0.05. ^a^All women aged 15–49 is used as the denominator

Prevalence of diarrhea and stunting has decreased between 2005 and 2011 survey years (Table 1). The concentration indices for all morbidities are negative, indicating a higher burden among children from poor households. The inequality across wealth strata was highest for the prevalence of stunting. The excess risk of the poorest quintile relative to the wealthiest quintile for having Acute Respiratory Infection (ARI), diarrhea, fever or stunting is 22 %, 43 %, 30 % and 71 %, respectively. The inequality in the rate of stunting has widened over the period 2005–2011.

The last row of table 2 shows the values of health inequity indices, calculated as the difference between the actual (the unstandardized concentration indices presented as “Total” in the table) and the contribution of all need factors to the concentration indices. The contribution of need factors to concentration index is negative for SBA (−2.1 %) and modern contraceptives (−1.4 %) suggesting that if utilization of these services were determined by need alone it would be pro-poor. In our case, the contribution of need factors to concentration index and their effect on health inequity index is very low highlighting the difficulty to define need for the interventions included in the analysis.

**Table 2:** Decomposition of the concentration indices for access to selected maternal and child health interventions in Ethiopia, 2011

	Contribution of need and non-need factors to the concentration indices for access to MCH care							
	Medical treatment for Diarrhea		Skilled birth attendance		Measles immunization		Modern Contraceptive	
	Absolute	%	Absolute	%	Absolute	%	Absolute	%
Need factors								
Demographic	0.0002	0.1	−0.0079	−1.1	0.0012	1.4	−0.0038	−1.4
Health status	0^a^	0.0	−0.0072	−1.0	-	-	0.0000	0.0
Subtotal	0.0002	0.1	−0.0151	−2.1	0.0012	1.4	−0.0038	−1.4
Non-need factors								
Wealth index	0.0510	33.5	0.1162	15.9	0.0578	68.2	0.1605	58.4
Educational attainment (women)	0.0294	19.3	0.1779	24.4	0.0175	20.7	0.0449	16.4
Educational attainment (men)	0.0205	13.4	0.0510	7.0	−0.0033	−3.8	0.0021	0.8
Area of residence (rural)	0.0158	10.4	0.2984	40.9	−0.0019	−2.3	0.0265	9.7
Subtotal	0.1097	77.0	0.6434	88.2	0.0702	82.8	0.2341	85.2
Residual	0.0355	22.8	0.1015	13.9	0.0134	15.8	0.0443	16.1
Total	0.1523		0.7299		0.0847		0.2747	
Horizontal inequity index	0.1521		0.7450		0.0835		0.2784	
Adjusted R^2^	0.05		0.35		0.13		0.21	

Note: ^a^Omitted from the analysis as recent episode of diarrhea was highly correlated with medical care seeking

The health inequity index is positive for all interventions, indicating that for a given need, children and women from wealthier households make greater use of available services in Ethiopia. Decomposition of the concentration index shows that 47 %, 66 %, 76 % and 85 % of wealth-related inequality in access to SBA, medical treatment for diarrhea, modern contraceptive use, and measles vaccination respectively is explained by the direct effect of household economic status and by educational attainment of parents. Area of residence contributes to large proportion (41 %) of the inequality in access for SBA to the disadvantage of the rural households. The elasticity of SBA with respect to women’s age and number of births (by a woman in the last five years) were both negative indicating that with increasing maternal age and birth order, the probability of birth attendance by a skilled professional decreases. On the contrary, for women in their reproductive age, the probability of using modern contraceptives on average increases with women’s age.

In order to assess the role of PHC expansion on changes in inequality in the utilization of MCH services, we used data on type of facility for diarrhea treatment, source for modern contraceptives and place of delivery. Utilization of services for diarrhea treatment, modern contraceptives and facility delivery in Ethiopia, on average, has improved over the period 2005–2011. Government PHC facilities played the major role for the improvement (Table 3). The contribution of PHC facilities as a point of care for diarrheal treatment, as source of contraceptives and place of delivery rose from 67 %, 74 % and 32 % in 2005 to 74 %, 85 % and 47 % in 2011 respectively. The lower socioeconomic groups are more likely to seek government PHC facilities as a source of modern contraceptive, as indicated by the negative concentration and health inequity indices (see Table 3). Even though concentration and health inequity indices for diarrhea treatment are positive for 2005 and 2011, both have shown a significant pro-poor improvement over the period 2005–2011. For all services, those with high socioeconomic status are more likely to report a visit to private facilities and the gap in private care utilization across socioeconomic groups has widened over time.

**Table 3:** Wealth related inequality and inequity in health care service utilization for diarrheal treatment, modern contraceptives and place of delivery by type of facility

	2005				2011			
	% using the service	Contribution (%)	Concentration index (95 % CI)	Horizontal inequity index	% using the service	Contribution (%)	Concentration index (95 % CI)	Horizontal inequity index
Diarrhea treatment								
Government hospital	4.7	21.9	0.069 ((−0.220)–0.358)	0.085	0.7*	2.0	0.347 (0.048–0.645)	0.353
Government PHC^a^	14.3	66.5	0.191 (0.111–0.270)	0.197	23.6*	73.5	0.094 (0.022–0.167)	0.091
Private facilities	2.5	11.6	0.140 (0.030–0.277)	0.151	7.9*	24.4	0.262 (0.100–0.424)	0.274
Total	21.5	100			32.1	100		
Source of Modern contraceptive								
Government hospital	0.8	4.5	0.616 (0.336–0.897)	0.577	0.3*	1.7	0.484 (0.229–0.739)	0.485
Government PHC^a^	12.9	74.4	−0.080 ((–0.103)–(−0.057))	−0.083	15.9*	84.9	−0.071 ((−0.089)–(−0.054))	−0.071
Private facilities	2.7	15.7	0.171 (0.101–0.241)	0.183	1.9*	10.3	0.398 (0.269–0.528)	0.395
Other	0.9	5.4	0.095 ((−0.188)–0.186)	0.130	0.6^**^	3.1	0.359 (0.030–0.690)	0.373
Total	17.4	100			18.7	100		
Place of Delivery								
Government hospital	3.0	52.6	0.829 (0.761–0.896)	0.854	3.8*	37.8	0.792 (0.720–0.864)	0.815
Government PHC^a^	1.8	31.6	0.735 (0.637–0.834)	0.745	4.7*	47.1	0.670 (0.588–0.752)	0.702
Private facilities	0.9	15.8	0.487 (0.281–0.692)	0.433	1.5^**^	15.1	0.691 (0.575–0.809)	0.679
Total	5.7	100			10.0	100		

Note: *Indicate the p-value for trend over the period 2005 and 2011 is <0.01. ^**^Indicate the p-value for trend over the period 2005 and 2011 is >0.05. ^a^PHC denotes primary health care facilities that include health centers, health stations and health posts

## Discussion

Despite improvements in coverage of MCH services, the inequality by wealth quintile has remained persistently high in all surveys. Socioeconomic status, measured by a wealth index and parental educational attainment, were the main predictors of differences in utilization of MCH services and health outcomes in children under five years of age. Area of residence has been a significant contributor for the disparity in access to SBA.

Among the health service coverage indicators (2011 DHS), use of modern contraceptive methods was the most inequitably distributed interventions, with a horizontal inequity index of 0.28. The average concentration index for 54 countdown countries for family planning needs satisfied was 0.14 (IQR: 0.05–0.2), making Ethiopia one of the countries with the most unequal distribution of the service [16]. Wealth level and educational attainment of women are estimated to jointly contribute to 75 % of this inequity in use of contraceptive methods. Several studies have demonstrated wealth and parental educational attainment as major determinants of access to MCH services in Sub-Saharan African countries [24, 25].

Albeit the low coverage of measles immunization in Ethiopia, it was the most equitably distributed indicator with a horizontal inequity index of 0.08 in 2011 DHS and it has shown a significant pro-poor improvement in comparison to 2005 DHS finding. The pro-poor improvement in measles immunization might be related to the “follow-up” measles vaccination campaigns conducted in Ethiopia. The low measles immunization coverage with marked heterogeneity by geographic location threatens the goals set out for elimination of measles at national and global levels [26].

The PHC service in Ethiopia is organized to deliver a package of basic preventive and curative health services targeting rural households. It is comprised of the following four health subprograms that conform to the elements of PHC as defined in the Alma Ata Declaration [27]: hygiene and environmental sanitation, disease prevention and control, health education and communication and family health (that include MCH, vaccination and family planning services).

PHC facilities have played an increasingly important role as points of care for diarrhea treatment and as a source of modern contraceptive for the less privileged socioeconomic group. Several studies have documented the effect of a scale up and equitable distribution of primary health care infrastructure and intervention coverage on inequality in service utilization and child health outcomes among different socioeconomic groups [23, 28, 29]. The role of PHC facilities as points of delivery care services in Ethiopia is relatively low. Public hospitals and private facilities play a major role as delivery care services outlet, more so for the wealthiest quintile and urban residents. The low utilization of these services among the poor and rural residents might be related to out-of-pocket spending by families, either for services or because families need to travel to a health facility. In countries where maternity hospitals are accessible and free of charge, coverage for SBA is almost universal [16]. Quality of care is an important aspect in utilization of delivery care services. The 2008 National baseline assessment for emergency obstetric and neonatal care has identified critical gaps in the delivery of quality obstetric and neonatal care in Ethiopia [30]. A study conducted in Ethiopia has also shown that women in rural Ethiopia strongly preferred health facility attributes indicative of good technical quality, reliable supply of medicines, functioning equipment and respectful provider attitude in selecting a delivery facility [31]. MCH services are among those services that suffer from inadequate resource allocation compromising delivery of quality services [10]. Cultural factors also influence utilization of facility delivery care service. According to 2011 Ethiopian DHS, 31 % of rural women reported that facility deliveries were not customary [9].

This study has some limitations. Recall bias is one possible problem in surveys as they are based on maternal recall. Differential reporting by rich and poor mother’s and between urban and rural residents is also a concern for a possible bias. The other limitation is that associated with asset indices. We have observed that the wealthiest quintile tend to reside in urban areas, particularly in the capital city, so that wealth inequities are closely associated with urban/rural disparities. In our analysis, the contribution of need factors to the horizontal inequity index was negligible. This could lead to a biased measurement of horizontal inequity index if there were other need factors (which we failed to include) that vary with income. Additionally, in the computation of concentration indices for binary outcomes, we used a linear regression model that may lead to inaccuracies.

Despite these limitations, our study adds important findings to the existing body of literature. The study included critical MCH interventions (such as family planning) and morbidity outcomes (for example, stunting) not addressed elsewhere. More importantly, we tried to assess if PHC expansion had any effect on inequality and inequity in access to care. The expansion of PHC facilities seems to have contributed positively to the coverage changes and the pro-poor and pro-rural improvements even though other factors (such as women’s education, safe water supply, food security) might have contributed as well. The 2008 World Health Report has reaffirmed the role of PHC as a pathway to achieve UHC and as a core strategy for health systems strengthening [32]. The new global investment frame work for Women’s and Children’s Health [33] has shown the substantial economic and social benefits of investing in Reproductive, Maternal, Neonatal and Child Health interventions. Nearly half of the reduction in child and maternal deaths was estimated to result from greater access to contraceptives for effective family planning that can be scaled-up at a relatively small cost using PHC as a delivery platform. The expected demographic dividend from the reduction in unintended pregnancy was estimated to exceed 8 % of the Gross Domestic Product by 2035 in countries with high fertility rate like Ethiopia. Further reduction in maternal and child mortality requires ensuring a reliable access to an integrated antenatal, intrapartum and postpartum care by skilled attendants [33, 34].

## Conclusions

While great progress has been made in Ethiopia, this analysis demonstrates that there is continued room for improvement to address persistently high inequality across the socio-economic spectrum. Future plans should aim to sustain current successes in health system strengthening and to bring these benefits to all women and children, particularly to those socioeconomically marginalized and rural residents. In addition to continued improvements to Ethiopia’s health sector, investments in women’s education and implementing pro-poor policies will be critical to maximize equitable health gains and population wide benefits. Monitoring the progress of intervention implementation should have an equity perspective.

## Abbreviations

ARI: acute respiratory infection
CI: concentration indices
DHS: demographic health survey
HCs: health centers
HEWs: health extension workers
HPs: health posts
HSDP: health sector development program
MCH: maternal and child health
PHC: primary health care
SBA: skilled birth attendant
UHC: universal health coverage
